# Three-Dimensional Neuroepithelial Culture from Human Embryonic Stem Cells and Its Use for Quantitative Conversion to Retinal Pigment Epithelium

**DOI:** 10.1371/journal.pone.0054552

**Published:** 2013-01-24

**Authors:** Yu Zhu, Madalena Carido, Andrea Meinhardt, Thomas Kurth, Mike O. Karl, Marius Ader, Elly M. Tanaka

**Affiliations:** Center for Regenerative Therapies, Technical University Dresden, Dresden, Germany; Purdue University, United States of America

## Abstract

A goal in human embryonic stem cell (hESC) research is the faithful differentiation to given cell types such as neural lineages. During embryonic development, a basement membrane surrounds the neural plate that forms a tight, apico-basolaterally polarized epithelium before closing to form a neural tube with a single lumen. Here we show that the three-dimensional epithelial cyst culture of hESCs in Matrigel combined with neural induction results in a quantitative conversion into neuroepithelial cysts containing a single lumen. Cells attain a defined neuroepithelial identity by 5 days. The neuroepithelial cysts naturally generate retinal epithelium, in part due to IGF-1/insulin signaling. We demonstrate the utility of this epithelial culture approach by achieving a quantitative production of retinal pigment epithelial (RPE) cells from hESCs within 30 days. Direct transplantation of this RPE into a rat model of retinal degeneration without any selection or expansion of the cells results in the formation of a donor-derived RPE monolayer that rescues photoreceptor cells. The cyst method for neuroepithelial differentiation of pluripotent stem cells is not only of importance for RPE generation but will also be relevant to the production of other neuronal cell types and for reconstituting complex patterning events from three-dimensional neuroepithelia.

## Introduction

The nervous system develops via formation of the neural tube — a pseudostratified epithelium with the apical cell surface oriented toward a single, central lumen. To reconstitute efficient and faithful differentiation of pluripotent cells toward neural cell types, we sought to reconstitute from pluripotent stem cells *in vitro* organized, three-dimensional neuroepithelial structures with a single lumen. We aimed to mimic the *in vivo* extracellular matrix environment by applying cell culture methods originally developed for polarized mammary and kidney epithelial cysts [1], [2]. Methods for differentiating embryonic stem cells (ESCs) via embryoid bodies or cell aggregates that undergo self-organization have made ground-breaking, fundamental contributions to our understanding of cellular differentiation and represent a valuable source of hard-to-obtain, differentiated cell types [3], [4], [5], [6], [7]. Such cultures often contain multiple cell types, so the desired target cell type must somehow be selected. Current two-dimensional (2D) conditions, on the other hand, can only generate homogeneous populations of only very few neuronal cell types [8].

Several degenerative diseases of the retina and the retinal pigment epithelium (RPE) have been characterized where transplantation may realistically ameliorate symptoms [3], [9], [10], [11], [12], [13]. Current methods to differentiate pluripotent cells to RPE include 2D differentiation of primate ESCs, spontaneous differentiation of colonies in human ESC cultures, as well as several floating aggregate methods using mouse and human ESCs (Table S1) [3], [4], [5], [14]–[20]. While several of these recent methods have significantly improved yield and accelerated differentiation, all methods to date result in a mixture of RPE cells and neural retina cells, thus requiring selection prior to RPE transplantation. The only method for RPE selection so far described has been manual picking and expanding pigmented colonies, limiting the relevance for large scale screening approaches and timely transplantation.

Here we demonstrate that a three-dimensional (3D) epithelial cyst culture of human pluripotent stem cells leads to the induction of polarized neuroepithelia within 5 days. This approach reconstitutes the 3D architecture of embryonic pseudostratified epithelium and the formation of a single lumen. We demonstrate the utility of this system by achieving quantitative production of RPE cells from human ESCs within 30 days. Direct transplantation of this RPE into a rat model of retinal degeneration without any selection and further expansion of the cells results in the integration of a RPE monolayer that rescues degeneration of the outer nuclear layer. Our work highlights how considering the cell biological context of pluripotent stem cells while culturing can significantly improve differentiation and the subsequent efficacy of therapeutic outcomes.

## Results

### hESC-derived cysts are composed of polarized neural progenitors

**Figure 1:**
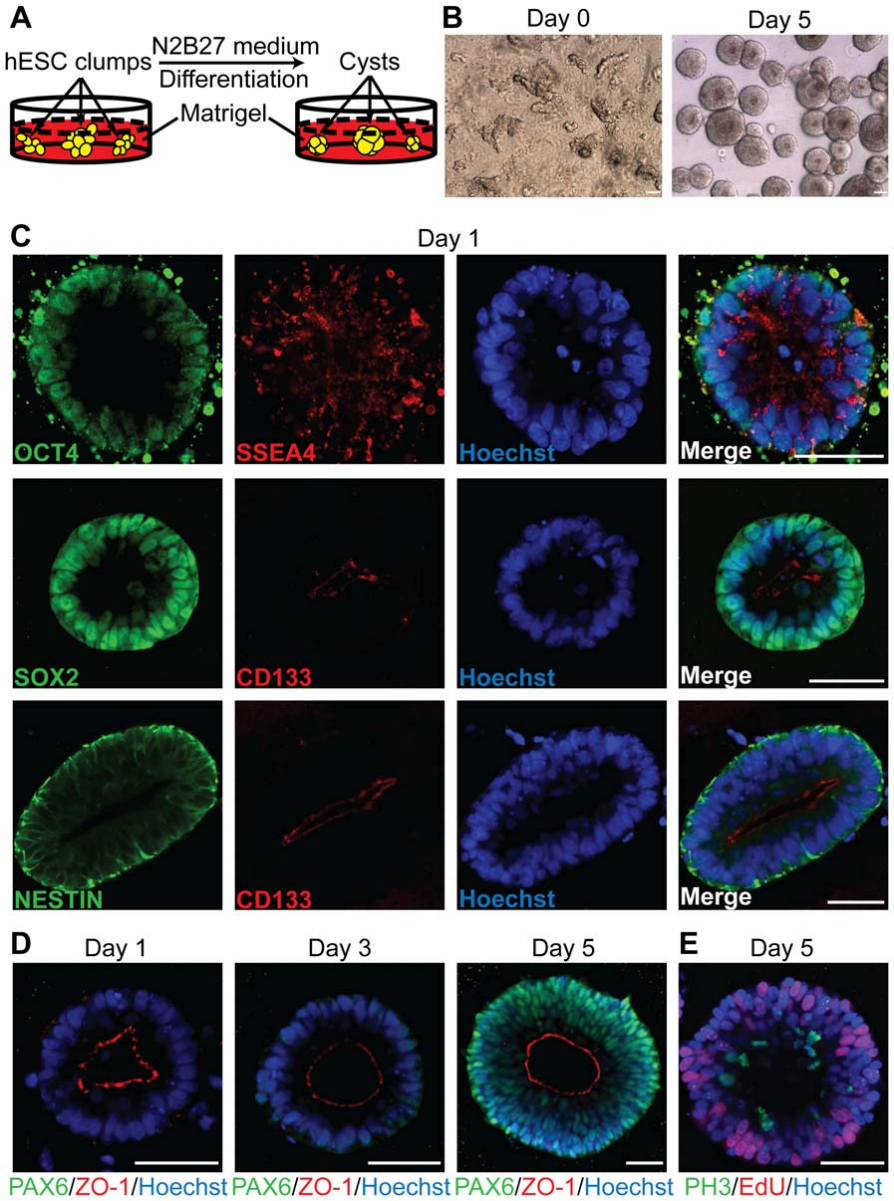
Efficient generation of polarized neural progenitors from hESCs in a Matrigel-based 3D neuroepithelial cyst model. (A) Schematic of the experiment. (B) hESC clumps found at Day 0 formed neural tube-like cysts with a single lumen by Day 5. (C) By Day 1, hESC-derived cysts were positive for SOX2 and NESTIN. The apical localization of CD133 indicates apicobasal polarity is firmly established. (D) Immunostaining of PAX6 and ZO-1 during cyst growth in Matrigel. PAX6 was strongly expressed in Day 5 cysts that display clear properties of a pseudostratified epithelium. (E) M-phase cells stained with Phospho-Histone H3 (PH3) antibody only localized at the apical side of the cysts and S-phase cells labeled with EdU at the basolateral side, indicating that luminal mitosis occurred within the cysts. Nuclei were counterstained with Hoechst. Scale bar, 50 µm.

To induce hESCs to faithfully reproduce neuroepithelial cell architecture, we embedded hESC clusters in the proteinaceous matrix Matrigel that had been reported to support 3D epithelial cyst formation [21] in the presence of the neural induction medium N2B27 (Figure 1A) [22]. Within 24 hours, all hESC clumps organized into neural tube-like structures with a smooth basal edge and an apical lumen (Movie S1, Figure 1B). We refer to these pseudostratified neuroepithelial structures as cysts, since they have a single lumen, similar to kidney and mammary epithelial cysts. The presence of a single luminal structure also distinguishes them from embryoid bodies or floating aggregates [3], [4], [23], [24]. Immunostaining of whole-mount preparations followed by confocal microscopy showed that hESC-derived cysts were composed of polarized neural progenitors. At Day 1, all formed cysts expressed pluripotency-related genes OCT4, SSEA4 and SOX2, as well as neural lineage-associated gene NESTIN (Figure 1C). The neuroectodermal marker PAX6 was gradually up-regulated so that it was strongly expressed by Day 5 in nearly all cysts, confirming a neural lineage (Figure 1D). The apicobasal polarity of the cysts was reflected by the apically restricted distribution of CD133 and ZO-1 at apical junctions from Day 1 on (Figure 1C and 1D). Apicobasal polarity was further demonstrated by luminal cell mitosis (Figure 1E), which is an important feature of polarized neuroepithelial cells. These observations indicate that the Matrigel-based 3D cyst model supports efficient generation of polarized neural progenitors from hESCs within 5 days.

### hESC-derived cysts acquire retinal identity

**Figure 2:**
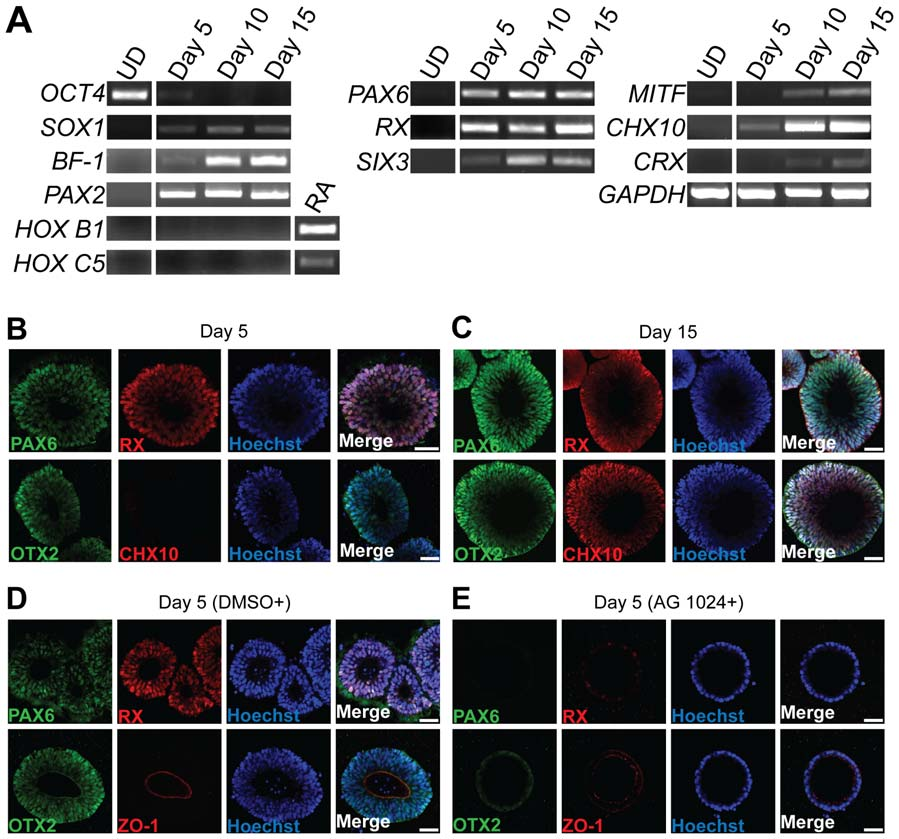
hESC-derived cysts enter and maintain retinal identity. (A) RT-PCR analyses for genes involved in retinal identity on undifferentiated hESCs (UD) and hESC-derived cysts at representative time points. Retinoic acid (RA) treated cysts were used as positive control for *HOX B1* and *HOX C5*. (B) Immunocytochemical analysis of hESC-derived cysts at Day 5. Upper row: cysts stained for PAX6 (green) and RX (red). Lower row: cysts stained for OTX2 (green) and CHX10 (red). Day 5 cysts were positive for PAX6, RX and OTX2 but few CHX10^+^ cells were detected at Day 5 indicating that cells achieved an optic vesicle identity. (C) Immunocytochemical analyses of hESC-derived cysts at Day 15. Upper row: cysts stained for PAX6 (green) and RX (red). Lower row: cysts stained for OTX2 (green) and CHX10 (red). CHX10^+^ cells were detected at Day 15. (D and E) Involvement of IGF-1/insulin signaling in optic vesicle stage neuroepithelium formation during hESC differentiation in Matrigel. hESC-derived cysts at Day 5 were co-stained with PAX6 and RX antibodies (upper row) or OTX2 and ZO-1 antibodies (lower row). Nuclei were counterstained with Hoechst. Scale bar, 50 µm.

We next identified the regional identity of hESC-derived cysts. During 2D *in vitro* hESC differentiation, hESC-derived neuroectodermal cells adopt anterior neuroepithelial characteristics in the absence of extrinsic patterning cues [24], [25]. Consistent with an anterior phenotype observed in other protocols, the cysts were *BF-1* and *PAX2* positive, as well as *HOX B1* and *HOX C5* negative (Figure 2A). Since expression of *BF-1* and *PAX2* suggests a possible retinal identity [26], [27], we examined the expression of eye field transcription factors (EFTFs), including *PAX6*, *RX* and *SIX3* [*28*]. All three transcripts were up-regulated from Day 5 (Figure 2A). When cysts were further cultured in Matrigel, the RPE-specific transcription factor *MITF* and the neural retina-associated transcription factor *CHX10* were strongly expressed from Day 10, suggesting that by Day 10 the cultures represented a mixture of RPE and neural retina-biased cells. Weak expression of *CRX*, which is highly expressed in photoreceptor precursors, was also detected from Day 10 (Figure 2A). Taken together, human ESC-derived cysts formed in Matrigel supplemented with neural induction media naturally express retinal progenitor genes as a population.

To quantitatively assess the efficiency of retinal induction within our cyst cultures, we immunostained the cysts for the optic vesicle stage-related markers PAX6, RX, OTX2 and the optic cup stage-related marker CHX10 (Figure 2B and 2C). The specificity of each antibody was verified on cryosections of appropriately staged embryonic mice (Figure S1). By Day 5, nearly all of the human ESC-derived cysts were PAX6^+^ (98.0±3.3%), OTX2^+^ (96.3±3.2%), and RX^+^ (91.9±7.7%) (means±SD, n_independent experiment_ = 3, more than 100 cysts counted for each experiment using H9 cell line). Within the positive cysts, a nearly uniform expression of PAX6, OTX2, and RX was observed. CHX10 was rarely expressed in Day 5 cysts (Figure 2B). At Day 15, most of the cysts maintained expression of PAX6, RX, and OTX2 while also becoming CHX10^+^ (61.9±4.6%) (means±SD, n_independent experiment_ = 3, more than 100 cysts counted for each experiment using H9 cell line) (Figure 2C). To confirm the results of the antibody staining, we performed *in situ* hybridization to detect the corresponding messenger RNA of cryosectioned cysts. These cryosectioned cysts showed strong signals for *PAX6* and *RX*, especially apically, (Figure S2A) suggesting that the stronger basal signal in the whole-mount immunofluorescence of large cysts (Figure 2B and 2C) might be due to the hindered penetrance of the antibodies into the center of the cyst.

We conclude from these results that the vast majority of hESC-derived cysts at Day 5 in the 3D differentiation model acquire an optic vesicle cell phenotype. Different batches of Matrigel may vary in the efficiency of generating optic vesicle stage cells. We tested three different lots of Matrigel, two of which showed no significant difference in generating PAX6^+^RX^+^OTX2^+^ cysts. The third lot of Matrigel showed weaker PAX6 induction in formed cysts.

### Retinal cyst formation involves IGF-1/insulin signaling

Our results indicate that growth in Matrigel alone with N2B27 medium is sufficient to induce retinal neuroepithelium from hESCs. Matrigel represents a mixture of not only extracellular matrix components but also soluble factors. In previous 2D induction protocols, IGF-1 (10 ng/ml) contributed to directing hESCs to a retinal progenitor identity [7]. Our Matrigel-based 3D model could potentially favor the retinal differentiation of hESCs due to the levels of IGF-1 reported to be in Matrigel (15.6 ng/ml) (www.bdbiosciences.com/external_files/dl/doc/manuals/live/web_enabled/356230_Guidelines.pdf). To test the possible role of IGF-1, we added the pharmacological inhibitor, AG 1024 that inhibits insulin receptor kinase and insulin-like growth factor-1 receptor kinase signaling, to the N2B27 medium at Day 0. In the presence of AG 1024, the percent of PAX6^+^ cysts at Day 5 was reduced from 98% to 33.9±3.3%. The PAX6^−^ cysts failed to achieve a pseudostratified architecture, and instead formed single cell layered epithelial cysts (Figure 2D and 2E). In addition, the expression of RX and OTX2 was also extremely low or absent in these PAX6^−^ cysts. ZO-1 expression was still mainly seen at the apical-lateral border of the cysts indicating that cell polarity was preserved (Figure 2E). These results suggest that IGF-1/insulin signaling is required for the formation of a pseudostratified, retinal neuroepithelium during hESC differentiation in Matrigel.

### Quantitative differentiation of polarized retinal progenitors to RPE or neural retina

**Figure 3:**
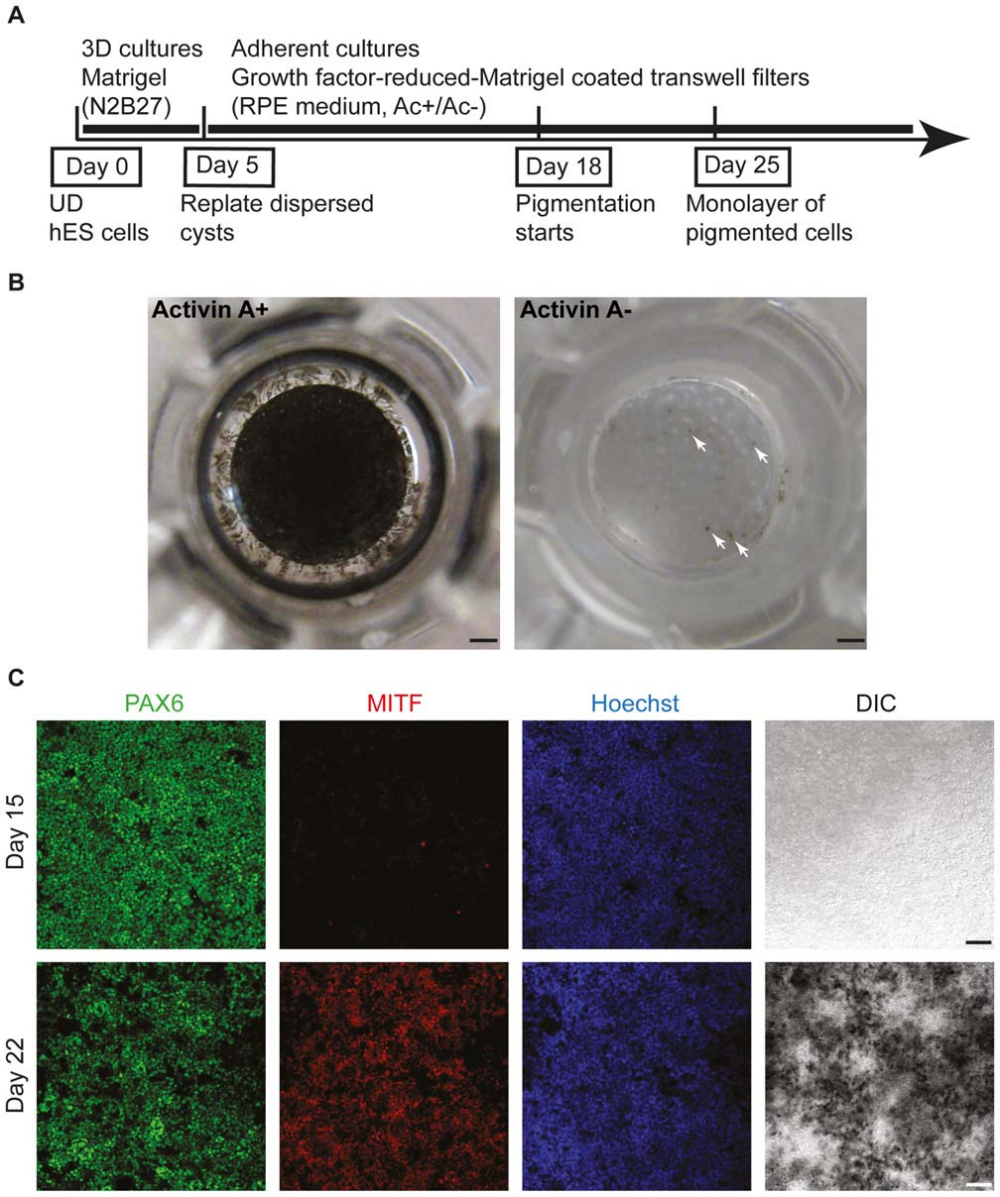
Directed differentiation of hESC-derived cysts to RPE using transwell filters. (A) Schematic of RPE differentiation protocol. UD: undifferentiated. Ac+/Ac−: with or without Activin A. (B) Top view of transwell filters at 30 days of culture showing the appearance of a pigmented cell sheet in the presence of Activin A (100 ng/ml) but not its absence. Arrows point to a few pigmented foci in the non-Activin A treated sample. (C) Immunostaining of PAX6 and MITF at Day 15 and Day 22 during RPE differentiation. Expression of PAX6 remained stable, while MITF was up-regulated by Day 22. Scale bars, 1 mm (B), 50 µm (C).

Given the high efficiency with which we could induce retinal progenitor cells, we sought to quantitatively differentiate these to RPE, one of the earliest differentiated retinal cell types during retinogenesis. Turning to another classical epithelial culturing method, we dispersed Day 5 cysts and plated them onto transwell filters coated with growth factor-reduced-Matrigel to obtain polarized epithelial cells under 2D culture conditions (Figure 3A). The culture medium was changed from neural induction medium (N2B27) to a standard RPE-supporting medium containing knockout serum replacement. In the presence of 100 ng/ml Activin A, a known inducer of RPE fate [29], we observed the appearance of pigmented cells starting at Day 18 and the characteristic polygonal cell shape emerging by Day 25. By Day 30, 95.7±1.0% of the cells were pigmented, whereas in the absence of Activin A only isolated colonies of pigmented cells appeared (Figure 3B and white arrows). To characterize the Activin A-dependent differentiation process, we performed a time-course analysis of some key features during RPE development (Figure 3C). Expression of PAX6 remained stable throughout the RPE differentiation process *in vitro*. The RPE-specific marker MITF started to be expressed in the majority of the cells by 22 days (Figure 3C). Importantly, Activin A-dependent RPE induction was highly sensitive to the initial cell seeding density on transwell filters (Figure S3A).

**Figure 4:**
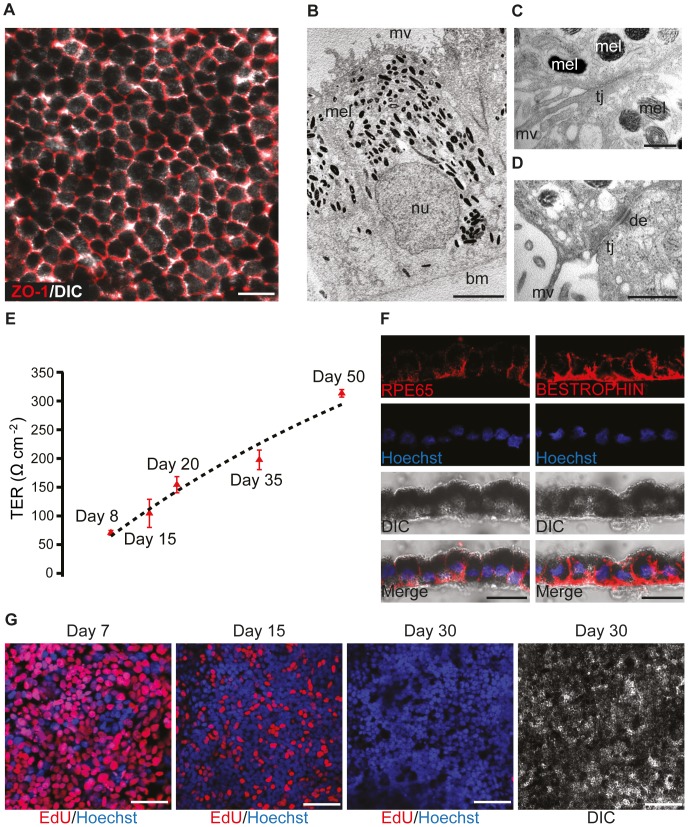
Characterization of the hESC-derived RPE sheets. (A) Immunostaining of ZO-1 (red) on hESC-derived RPE cells at Day 30 indicated the presence of tight junctions. Pigmented cells displayed polygonal shape. (B–D) Electron microscopic analyses of hESC-derived RPE cells at Day 50. hESC-derived RPE cells had abundant apical microvilli (mv), melanin granules (mel) in the apical half and the nucleus (nu) in their basal half. A basement membrane (bm) was visible. Tight junctions (tj) and desmosomes (de) could be found. (E) The transepithelial resistance (TER) of hESC-derived RPE progenitor or RPE cells increases during differentiation. (F) Cross-sections through the pigmented cell sheet immunostained for RPE65 and BESTROPHIN. hESC-derived RPE cells at Day 40 expressed the mature RPE cell markers RPE65 and BESTROPHIN. (G) hESC-derived RPE cells exited the cell cycle by Day 30 as demonstrated by RPE cultures pulse labeled with EdU at Day 7, 15 and 30. EdU incorporation by proliferating cells was observed at Day 7 and 15, but rarely observed at Day 30.

We assessed the differentiation of the RPE sheets post-Day 30 using a variety of molecular, ultrastructural, and functional assays. Several indicators showed that the cultures formed a tight epithelial barrier including robust ZO-1 immunofluorescence (Figure 4A). Ultrastructural analysis at Day 50 confirmed the presence of tight junctions, desmosomes, microvilli and abundant melanin granules enriched toward the apical surface, which displayed the characteristic dome shape (Figure 4B–4D). To functionally test the epithelial barrier function, we measured the transepithelial resistance (TER) which gradually increased to a TER of 313±4 Ω·cm^−2^ at Day 50 (Figure 4E). This value is within the upper range of TER measured from cultured human fetal RPE (150 and 330 Ω·cm^−2^) [30], [31], suggesting that the epithelial barrier function has been established. To further characterize RPE differentiation, we immunostained at Day 40 for mature RPE cell markers RPE65 and BESTROPHIN (Figure 4F). Finally, we confirmed that the proliferating RPE progenitor cells exited the cell cycle over time based on 5-ethynyl-2′-deoxyuridine (EdU) uptake at representative time points. As shown in Figure 4G, the vast majority of pigmented cells were negative for EdU by Day 30.

We asked whether the Day 5 retinal progenitor cells could alternatively be differentiated into neural retina cells with similar efficiency and speed. Plating of cysts onto transwell filters and further culturing in neural induction medium rather than RPE medium (Figure S4A) resulted in a uniform culture of CHX10^+^PAX6^+^ cells where >99% of cells were CHX10^+^PAX6^+^ by 20 days (Figure S4B). The expression of CRX on protein level was not detectable by 20 days, although weak *CRX* expression was detected in RT-PCR (Figure 2A) by 10 days of differentiation. By 40 days post induction, rosette-like structures formed on the transwell filters with the appearance of a small number of CRX^+^ cells. By 60 days, cells had self-organized into stratified, rosette-like structures with a high percentage of CRX^+^ cells surrounded by CHX10^+^PAX6^+weak^ and CHX10^−^PAX6^+strong^ cells, suggesting their neural retina cell identity. This stratified structure resembled the layered structure seen in the intact mouse retina (Figure S4C).

These findings demonstrate that the sequential 3D and 2D epithelial culture of hESCs efficiently directs them toward RPE or neural retina depending on the media conditions applied. From the same hESC-derived cysts we have generated either pigmented cells that have morphological properties highly characteristic of native RPE cells and form a confluent RPE cell layer without the need for clonal expansion or neural retina cultures that have a high percentage of CRX^+^ cells.

### Phagocytic function of hESC-derived RPE cells

**Figure 5:**
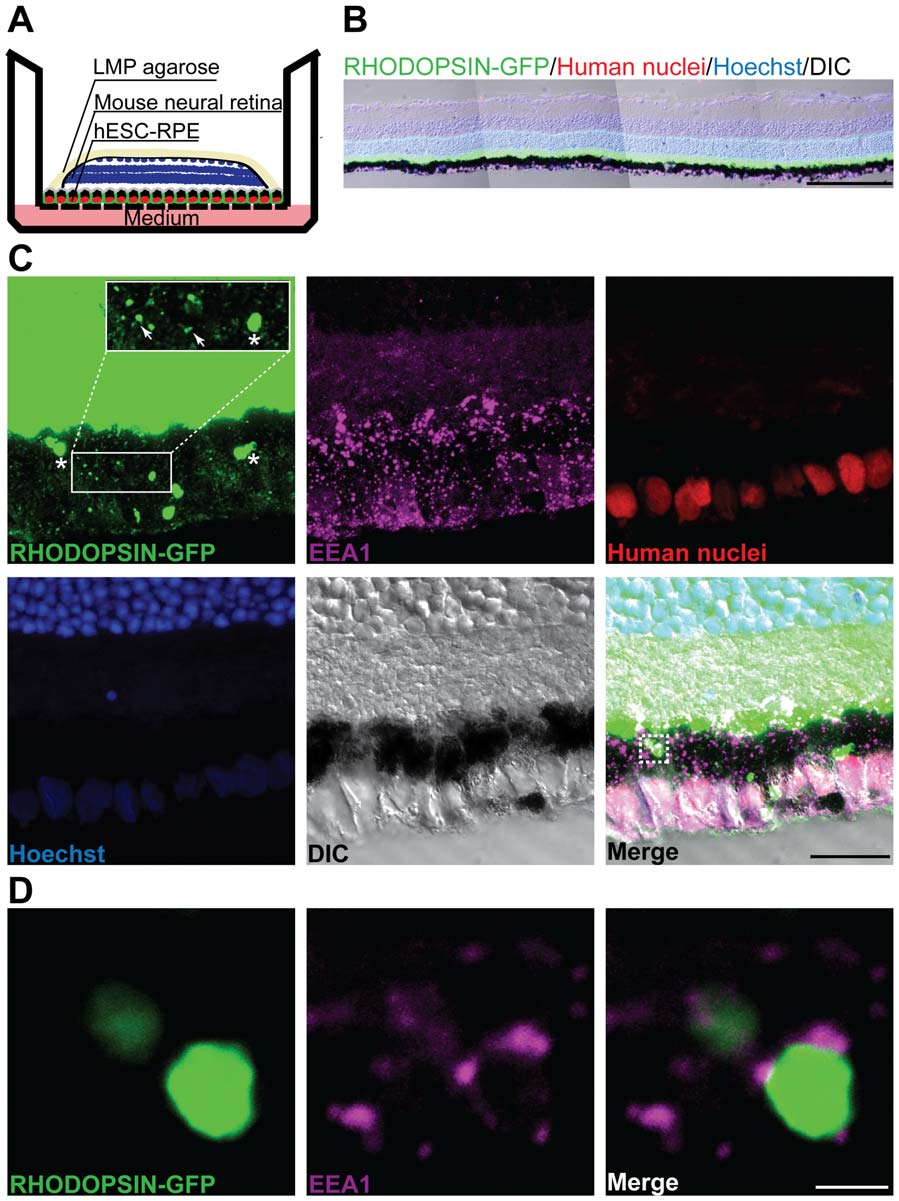
Co-culture of hESC-derived RPE cells and mouse retinal explants (RHODOPSIN-GFP fusion construct transgenic mice) *in vitro* to assess phagocytosis. (A) Schema of the *in vitro* co-culture model. LMP: low melting point. (B) A merged RHODOPSIN-GFP (green), human nuclei (red), Hoechst (blue) and DIC image in the co-culture model. hESC-derived RPE cells formed a contiguous tissue sheet with the mouse retinal explant. (C) Immunocytochemical analyses on cross-sections of co-cultured hESC-derived RPE cells and mouse retinal explants. Fluorescence images represent the maximum intensity projection of serial confocal images through z-axis. Pigmented layer cells were positive for antibody staining against human nuclei (red). Stars or arrows point to bigger or smaller pieces of GFP^+^ photoreceptor outer segments (green) found within hESC-derived RPE cells. Immunostaing for the early endosome membrane marker EEA1 using a human-specific antibody (magenta) is visible in the pigmented layer associated with phagocytosed GFP^+^ outer segments. Inset in Rhodopsin-GFP channel: higher magnification view of phagocytosed outer segments. (D) A high-magnification image of dashed box in the merge channel of panel (C) shows that the EEA1 signal surrounds the GFP^+^ particles. Nuclei were counterstained with Hoechst. Scale bars, 200 µm (B), 20 µm (C), 2 µm (D).

One of the most important functions of RPE cells *in vivo* is phagocytosis of shed photoreceptor outer segments, supporting the daily renewal of photoreceptor cells [32]. To analyze whether our hESC-derived RPE cells phagocytose shed outer segment disks, we endeavored to recapitulate the *in vivo* photoreceptor/RPE interaction by co-culturing the hESC-derived RPE cells with neural retinal explants from transgenic mice expressing human RHODOPSIN-GFP [33] (Figure 5A), similar to a previously described co-culture system [9]. We prefer this system over using purified outer segments or latex bead incubation assays where it can be difficult to distinguish truly endocytosed disks from material adherent to the surface. After 24 hours of co-culture, the hESC-derived RPE cells were directly juxtaposed with the mouse retina cells (Figure 5B). Immunofluorescence to specifically detect human nuclei in the pigmented cell layer excluded the possibility of contamination from mouse RPE cells. Abundant GFP^+^ outer segment structures were observed within the pigmented cells (Figure 5C, Movie S2). Immunostaining of co-cultures to detect the early endosome marker EEA1 specifically in human cells showed EEA1 signal surrounding GFP^+^ particles, suggesting that outer segments had been truly phagocytosed into the RPE cells (Figure 5D).

### Differentiation of RPE cells from other human pluripotent stem cell lines

The bulk of our analysis was performed on the hESC line H9, but we confirmed that our hESC-to-RPE protocol also worked efficiently on different human pluripotent cell lines. The data represented in Figures 1, 2, 3, and 4 were reproducible with the H1 cell line, except for the requirement of exogenous Activin A, as described below. In our H9 work, the addition of Activin A significantly enhanced the appearance of pigmented cells (Figure 3B) and efficient RPE induction was highly sensitive to cell seeding density in the presence of Activin A (Figure S3A). When using the H1 cell line, the RPE differentiation efficiency and timeline was very similar compared to that of the H9, but exogenous Activin A was not required for RPE differentiation. However, the pharmacological Activin/TGF-β inhibitor SB 431542 completely blocked pigmentation in H1 cells, indicating that Activin-like signaling is indeed required and is presumably made endogenously during RPE differentiation from H1 cells (Figure S3B).

We also determined if our method was effective on the human induced pluripotent stem cell (iPSC) line O27-08, which was reprogrammed from human foetal neural stem cells (CB660) [34]. The timescale of cyst formation and RPE differentiation were indistinguishable from those of hESCs (Figure S5A and S5B). During differentiation, immunostaining revealed that the human iPSC-derived cysts were also positive for retinal progenitor markers PAX6, RX and OTX2, with the tight junction marker ZO-1 toward the apical side (Figure S5C). However, we found Pax6 expression in O27-08 cell-derived cysts was weaker compared to that in hESC-derived cysts. This coincides with previous reports in which considerable variation was found in the ability of different iPSC cell lines to differentiate into PAX6^+^ neuroectodermal cells [16], [35]. Although the percentage of pigmented cells differentiated from O27-08 cells was lower compared to the H9 and H1 hESCs, we obtained a relatively high percentage of RPE cells in the 30-day timescale (48.0±6.2% pigmented cells), still representing a significant improvement to other RPE protocols using ESCs (Figure S5B, Table S1). It is likely that boosting PAX6 induction by other exogenous factors, or using iPSC cell lines that are biased toward RPE formation [36] might rescue this lower efficiency.

Collectively, these results demonstrate that the optic vesicle stage cells can be derived from different human pluripotent cell lines and can be further directed toward RPE cells with exogenous or endogenous Activin A signals.

### *In vivo* integration and function of transplanted RPE

We then determined if our RPE could be directly transplanted without manual selection into Royal College of Surgeons (RCS) rats to rescue retinal degeneration *in vivo*. RCS rats are the primary animal model currently used for RPE replacement studies. The rats are characterized by a phagocytosis defect in the RPE due to a mutation in the receptor tyrosine kinase Mertk gene [37]. This defect leads to an enrichment of outer segment fragments in the sub-retinal space. Accumulation of debris causes degeneration of photoreceptors with an almost complete loss of the outer nuclear layer (ONL) at an age of six weeks.

**Figure 6:**
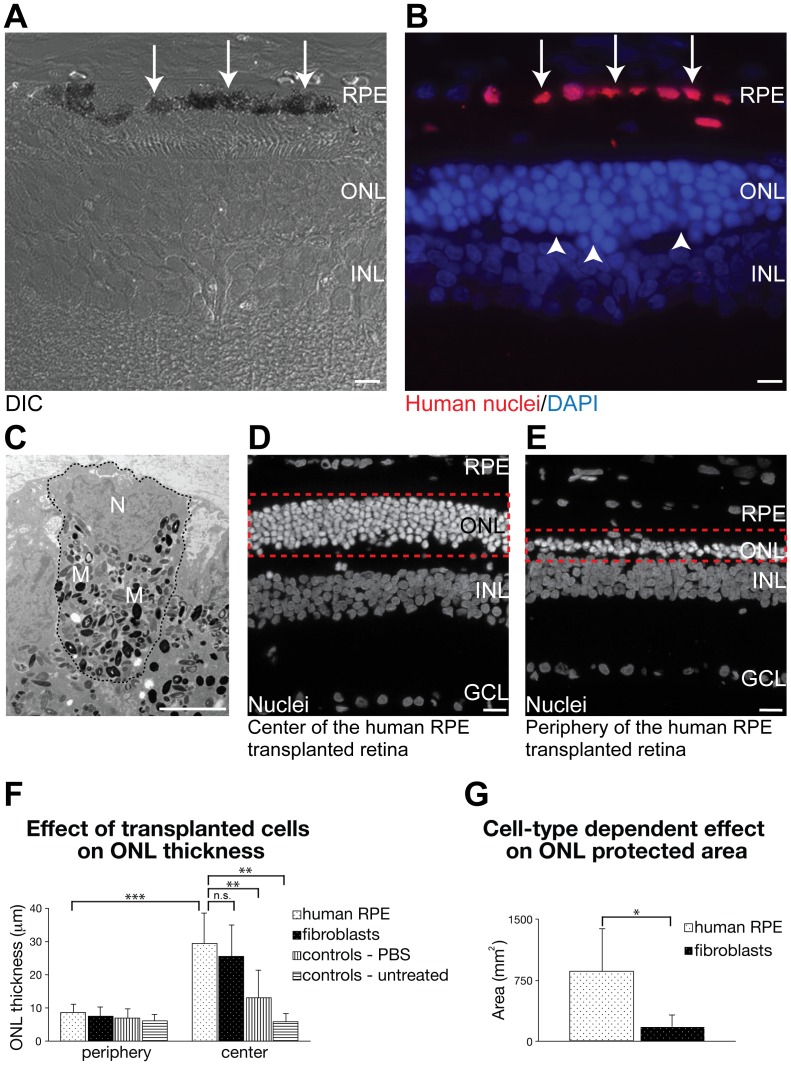
Transplantation of hESC-derived RPE cells effectively rescues photoreceptors in RCS rats. (A, B) Sections of transplanted retina in the central region adjacent to the injection site shown in DIC image (A) and immunostained to identify human nuclei (red) and DAPI (blue) (B). Donor RPE cells (red) were localized in the sub-retinal space integrated into the host RPE monolayer and generated monolayer-like structures (A, B, arrows). The ONL overlying the donor RPE monolayer was well preserved and contained 5–6 rows of nuclei (B, arrow heads). (C) Electron microscopic analyses of transplanted RPE cells. The dotted line outlined one RPE cell. Typical polarized RPE morphology with foldings at the basal membrane, a nucleus located at the basal side (labeled by N) and apically located melanin-containing melanosomes (some labeled by M) were observed. (D, E) Representative fluorescence images of central or peripheral region in the hESC-derived RPE cell transplanted retina. Dashed box showed the ONL. Following transplantation of donor RPE cells, central regions of the retina showed an increased ONL thickness with an average of 5–6 rows of nuclei (D) in contrast to the periphery with 1–2 rows of nuclei (E). (F) Quantitative data for the effect of different transplanted cells on ONL thickness. (G) Quantitative data for the protected area of ONL in the human RPE and fibroblast cell transplanted retina. In panels (F) and (G), 6 RCS rats transplanted with human ESC-derived RPE cells, 5 with human fibroblasts, 5 sham-injected, and 6 untreated controls were quantified. Data were presented as means±SD. GCL: ganglion cell layer, INL: inner nuclear layer, ONL: outer nuclear layer. n.s.: not significant, *: P<0.05, **: P<0.01, ***: P<0.001. Nuclei were counterstained with DAPI. Scale bars, 10 µm (A, B), 5 µm (C), 20 µm (D, E).

Human H1 and H9 ESC-derived RPE cells were transplanted into the sub-retinal space of 19–21 day-old RCS rats and identified by human cell-specific markers, i.e. human nuclei and β2-microglobulin, 5–6 weeks after grafting (Figure 6A, 6B, Figure S6A). Directly at the injection site, human ESC-derived RPE cells were found in clusters and obvious disruption of the retinal structure was observed (Figure S6A). However, next to the injection site, the donor RPE formed a monolayer with direct contact to adjacent outer segments (Figure 6A, Figure S6A). Correspondingly, the ONL structure overlying the donor RPE layer was well preserved (Figure 6B, Figure S6A). Apical-basolateral polarization of the transplanted RPE cells was confirmed at the ultrastructural level with a basally located nucleus and apically distributed melanosomes containing melanin (Figure 6C). Additionally, electron-microscopic analysis of junctions between human ESC-derived RPE cells, identified by pigmentation and morphology, showed the formation of tight junctions and/or desmosomes between donor cells (Figure S6B), a typical feature of endogenous RPE monolayers.

To determine the extent of ONL rescue, we compared ONL thickness in regions with organized structure overlying transplanted RPE monolayers, versus peripheral regions lacking transplanted cells. Fluorescence images of tissue sections revealed that ONL associated with transplanted human ESC derived RPE contained 5–6 rows of nuclei (Figure 6D). In contrast, ONL in peripheral regions lacking transplanted cells contained 1–2 rows of photoreceptor nuclei (Figure 6E), similar to untreated control retinas (Figure S6C, S6D). The ONL thickness of experimental retinas (n = 6) was quantified and compared with untreated RCS rats (n = 6), animals injected with human fibroblasts (n = 5), or PBS (sham injection) (n = 5). In all experimental groups, the ONL in peripheral regions lacking transplanted cells displayed a thickness of ≈8–9 µm, corresponding to 1–2 rows of nuclei (Figure 6E). In contrast, in areas containing donor RPE the ONL was significantly thicker (29.70±9.34 µm) compared to untreated controls (5.91±2.38 µm) or sham-injected animals (12.99±8.39 µm) (Figure 6F). Interestingly, in accordance with previous studies that also demonstrated photoreceptor rescue following injection of several other, non-RPE cell-types [13], [19], transplantation of human fibroblasts had an almost similar protective effect on ONL thickness as human ESC-derived RPE cells reaching 25.38±9.49 µm in central regions of experimental retinas (Figure 6F).

To further evaluate the protective effects of RPE versus fibroblast transplantations, we quantified the total retinal area that showed increased ONL thickness for both experimental groups (Figure 6G). Although similar numbers of cells were transplanted, retinas that received human ESC-derived RPE showed a significantly increased area of rescued ONL (855.63±513.91 mm^2^ with a maximum of 1900 mm^2^) compared to human fibroblast recipients (178.90±148.85 mm^2^) (Figure 6G). Given that only the RPE cells were able to correctly integrate into the host RPE monolayer, these results suggest better functionality or survival of donor RPE cells over fibroblasts. A potential explanation for the improved rescue effect by donor RPE cells might be the production and secretion of RPE-specific factors that are essential for photoreceptor development, survival, maintenance and function. The identification of such factors will have to be evaluated in detail in future studies.

Taken together, our results indicate that RPE cells that were differentiated *in vitro* from neuroepithelial cysts and transplanted without any pre-selection efficiently form mature RPE *in vivo*. We observed no tumorigenesis within the timescale of our experiments.

## Discussion

This study demonstrates that the application of a Matrigel-based 3D epithelial cyst culture to human pluripotent stem cells results in an efficient reconstitution of neuroepithelial structures including the formation of a single lumen. Recently, mouse ESC aggregates cultured in laminin and Nodal were shown to form three-dimensional structures where a major part of the cluster organized into multi-layered optic vesicle-like tissue that formed neural retina and RPE; a ground-breaking system, important for studying complex, inductive events between different cell types [4]. Modified conditions placing floating aggregates in Matrigel plus WNT inhibitor, FBS, and SHH activators have also been utilized to obtain self-organizing retinal structures from human ESCs [6]. There are several important distinctions between such methods and the one we introduce here. In our system, pluripotent cells are not pre-aggregated, but rather directly placed in neural induction conditions in Matrigel. This leads to an unprecedented speed of neural induction compared to other methods [4], [23], [24], [38]. Human ESCs form a PAX6^+^ neuroepithelium within 5 days representing a significant enhancement in the speed of neural induction compared to previously described methods. In contrast to self-organizing embryoid bodies or neural aggregates, our human ES-derived cysts have a single lumen and are uniform in cellular identity. The uniformity and patternability of the neuroepithelia was evidenced in the human ESC system where cysts uniformly achieve retinal identity within 5 days. From these cysts we could quantitatively generate neural retina epithelium or RPE under directed culture media conditions, rather than relying on self-organization of the optic vesicle tissue. These experiments open the path for future experiments reconstituting neuroepithelial patterning directly in neuroepithelial cysts using defined soluble factors.

The RPE cells from our human ESC-derived cysts have morphological and functional properties highly characteristic of native RPE cells and, importantly, form a confluent RPE cell layer without the need for isolation and further expansion. Therefore our method provides an unprecedentedly high efficiency and speed of human RPE cell sheet generation, which could be applied toward drug screening, transplantation approaches for the replacement of RPE in retinal degeneration, and for studying human RPE biogenesis at the biochemical level. All previous protocols required manual sub-selection and expansion [3], [9], [14], [15], [18]. This is important since *in vitro* passaging of primary RPE cells can result in an epithelial to mesenchymal transition and loss of RPE phenotype [39].

Our *in vivo* experiments demonstrate that direct transplantation of hESC-derived RPE without selection or expansion resulted in integration and monolayer formation, confirming the robust epithelial structural integrity of these cells. The transplanted monolayers significantly rescued overlying photoreceptor cells from degeneration in RCS rats, the standard model for RPE transplantation studies. Whether the observed protection of the ONL is caused by a paracrine effect or phagocytic function of donor RPE has to be further evaluated given the finding that human fibroblasts showed similar effects on the ONL thickness of RCS rats. However, hESC-derived RPE protected a significantly larger area of ONL than fibroblasts, pointing to a superior effect of these cells. In light of recent, promising transplantation approaches for the replacement of dysfunctional or lost RPE in pre-clinical models [3], [9], [10], [12], [19] and human patients [11] with ES or iPS cell-derived RPE [40], [41], [42], this rapid protocol for directly differentiating pure, human RPE cell populations from an unlimited *in vitro* cell source has high medical relevance. Regarding the possibility of using such cells for human transplantation, further caution on the purity of transplanted RPE cells should be considered. Before use of these cells in clinical applications, their functionality following transplantation *in vivo* has to be further investigated to evaluate their potential for e.g. phagocytosis of photoreceptor outer segments or recycling of retinoids, besides analyzing their rescue capacity in other preclinical models characterized by RPE loss or dysfunction.

## Materials and Methods

### Ethics Statement

iPSC line O27-08 was generated from human foetal neural stem cells (CB660) which were derived with Ethical approval from the Lothian Research Ethics Committee (LREC/2002/6/15). Human cell lines were derived with written informed consent by the donors with respect to taking the samples and making the cell lines. All animal experiments were carried out in strict accordance with European Union and German laws (Tierschutzgesetz) and adhered to the ARVO Statement for the Use of Animals in Ophthalmic and Vision Research. All animal experiments were approved by the animal ethics committee of the TU Dresden and the Landesdirektion Dresden (approval number: 24-9168.11-1/2010-23).

### Maintenance of hESCs and human iPSCs

hESC lines H9, H1 (WISC Bank) [43] and one iPSC line O27-08 (gift of Yasuhiro Takashima and Austin Smith, University of Cambridge, Cambridge, UK) were cultured in mTeSR1 medium (StemCell Technologies) on BD Matrigel hESC-qualified Matrix (BD Biosciences)-coated plates [44]. The hESCs and human iPSCs expressed OCT4 and SSEA4 confirming the undifferentiated state. Cells were passaged every 5–6 days using Dispase (StemCell Technologies). Morphologically distinguishable differentiated cells were mechanically removed at each passage. To improve cell survival during passaging, the Rho kinase inhibitor, Y-27632 (Calbiochem), was added in the culture medium during the first 24 hours after plating. For storage, the hESC colonies were suspended in mFreSR™ (StemCell Technologies), frozen in an isopropanol freezing container at −80°C overnight and transferred into liquid nitrogen the next day.

### Differentiation of hESCs and human iPSCs in the neuroepithelial cyst model

Undifferentiated cell colonies were partially lifted by Dispase and scraped off with a cell scraper. Detached cell aggregates (diameter: 50–100 µm) from one confluent well of a six-well plate were embedded in 150–300 µl of Matrigel. 50 µl of Matrigel containing the cell clumps was added per square centimeter of growth surface. After gelling at 37°C for 10 min, cells embedded in the Matrigel layer were cultured in neural induction medium N2B27 which consisted of DMEM/F12+GlutaMAX™ (GIBCO), neurobasal medium (GIBCO), 0.5×B27 supplement (GIBCO), 0.5×N2 supplement, 0.1 mM β-mercaptoethanol and 0.2 mM L-Glutamine, prepared as described [22]. In separate experiments, AG 1024 (Calbiochem, 10 µM, from Day 0 to 5) or DMSO was added to differentiating hESC cultures.

### Differentiation of RPE or neural retina cells on transwell filters

hESC- or human iPSC-derived cysts at Day 5 were taken out of the Matrigel using Cell Recovery Solution (BD Biosciences) and treated with TrypLE™ (Invitrogen), followed by gentle trituration to achieve single cell suspension. To get rid of the few non-cyst cells, large cysts were permitted to sink down to the bottom of the tube by gravity. 40 µm-cell strainer (BD Biosciences) was used to exclude big clumps after TrypLE™ treatment. Dispersed cells were resuspended in N2B27 medium and plated onto growth factor-reduced-Matrigel (BD Biosciences)-coated 6.5 mm Transwell® with 0.4 µm Pore Polyester Membrane Insert (Corning Costar) at a density of 2–4×10^5^ cells/well. At Day 6, attached cells were washed twice with RPE medium-DMEM+GlutaMAX™ medium supplemented with 20% knockout™ serum replacement (Gibco), Non-Essential Amino Acids, 1 mM L-Glutamine and 0.1 mM β-mercaptoethanol. The cells were then kept in RPE medium with or without 100 ng/ml human Activin A (Peprotech). In the experiments of inhibiting TGF-β signaling, SB 431542 (Sigma, 8 µM) was added in cultures from Day 6 to 25. For neural retina differentiation, the cells were kept in N2B27 medium when culturing on transwell filters. The medium was changed every 2–3 days.

### Animals and transplantation

Host animals were 19 to 21 days old RCS albino rats, which were maintained in a 12 h light/dark cycle, with food and water *ad libitum*. For transplantation, human ESC-derived RPE cells were washed with PBS and incubated 20 minutes at 37°C with 0.5 mg/ml trypsin (Invitrogen). Then 2 mg/ml trypsin inhibitor (Roche Diagnostics) and 0.1 mg/ml DNase I (Invitrogen) were added. Additional mechanical dissociation was performed. The cells were centrifuged 5 minutes at 300 g and finally resuspended in PBS.

Animals were anesthetized by an intraperitoneal injection of medetomidine hydrochloride (0.01 mg/10 g body weight, Dormitor; Pfizer) and ketamine (0.75 mg/10 g body weight, Ratiopharm) and fixed in a head holder. Pupils were dilated by drops of 1% tropicamide (Mydrum, Dr. Mann Pharma GmbH) and 2.5% phenylephrine (TU Dresden Pharmacy). One to three microliter of cell suspension containing ≈60,000 to 130,000 cells were injected into the subretinal space using a syringe (Hamilton, Reno, NV) with a blunt, 34-gauge needle via a trans-vitreal approach (n = 10). Control eyes were transplanted with human fibroblasts (Lonza) (n = 5), sham-injected (PBS) (n = 8), or untreated (n = 7). For recovery, experimental animals received an injection of atipamezole hydrochloride (0.1 mg/10 g body weight, Antisedan) for reversal of the medetomidine.

### Immunocytochemistry

Cells or cysts were fixed with freshly made 4% paraformaldehyde (PFA) for 20 min at room temperature (RT), followed by antigen retrieval (if necessary, Citrate buffer, 70°C, 30 min), quenching (1×PBS, 100 mM Glycine, 0.3% Triton X-100) and blocking (1×PBS, 0.5% BSA, 0.3% Triton X-100). Immunostaining was performed in blocking buffer and each antibody reaction was done overnight at 4°C. The primary antibodies and their working dilutions were as follows: rabbit anti-NESTIN (1∶500, Abcam), rabbit anti-SOX2 (1∶100, Zymed), mouse anti-CD133 (1∶50, Miltenyi Biotec), rabbit anti-ZO-1 (1∶100, BD Biosciences), rabbit anti-PAX6 (1∶200, HISS Diagnostics), mouse anti-PAX6 (1∶20), rabbit anti-OTX2 (1∶200, Abcam), sheep anti-CHX10 (1∶200, Chemicon), mouse anti-MITF [C5] (1∶50, Abcam), mouse anti-RPE65 (1∶500, Millipore), mouse anti-BESTROPHIN (1∶1000, Millipore), mouse anti-human nuclei (1∶50, Millipore), mouse anti-EEA1 (1∶1000, gift of Marino Zerial, Max Planck Institute of Molecular Cell Biology and Genetics, Dresden, Germany), and goat anti-GFP (1∶400, Rockland). Antibody against PAX6 was obtained as previously described [45]. Antibodies against RX and CRX were raised in rabbits immunized with a portion of human RX (residues 216–258) or human CRX (residues 112–259) tagged with GST and affinity-purified against the corresponding MBP-RX or MBP-CRX fusion proteins respectively. The immunoreactivity of each antibody was confirmed by immunostainings on mouse tissues of corresponding stage as a positive control. The secondary antibodies used were as follows: Alexa Fluor 488 donkey anti-rabbit IgG, Alexa Fluor 647 donkey anti-rabbit IgG, Alexa Fluor 555 donkey anti-mouse IgG, Alexa Fluor 555 donkey anti-sheep and Alexa Fluor 488 donkey anti-goat IgG (1∶200, Invitrogen).

For EdU incorporation, hESC-derived cysts at Day 5 were incubated with EdU for 1 hour, while RPE preparations on transwell filters were incubated with EdU for 24 hours. Click-iT™ EdU Alexa Fluor® 647 Imaging Kit (Invitrogen) was used for detection.

Cell nuclei were counterstained with Hoechst 33342 (1–5 µg/ml, Sigma). Cells were imaged using a Zeiss LSM 510 confocal microscope, Leica TCS SPE confocal microscope or Zeiss Axiovert 200 microscope.

### RT-PCR analyses

Total RNA was extracted from undifferentiated or differentiating hESCs using a RNeasy kit (Qiagen), treated with RNase-free DNase I (Invitrogen) and reverse-transcribed with SuperScript II (Invitrogen). The synthesized cDNA was amplified with gene-specific primers. PCR was carried out with Taq DNA Polymerase with denaturation 94°C–20 s, annealing 55°C–30 s, extension 72°C–60 s for 35 cycles. The primers used are available in Table S2.

### Electron microscopic analyses

Cells were fixed and processed on the transwell filters. They were fixed with 2% glutaraldehyde and 2% PFA in 50 mM HEPES overnight at 4°C. After several washes in 100 mM HEPES and PBS they were postfixed with 1% OsO_4_/PBS for 2 hours on ice, washed with PBS and water, and *en bloc* contrasted with 1% uranyl acetate in water, again for 2 hours on ice. Samples were dehydrated in a graded ethanol series, infiltrated in ethanol/epon mixtures (1∶3, 1∶1, 3∶1) and in pure epon (2×) and cured at 60°C for 24 hours. Samples were cut to small pieces (1–2 mm), remounted for cross-sectioning of the RPE layer on empty epon dummy blocks and cured for another 24 hours at 60°C. Ultrathin sections were stained with lead citrate and uranyl acetate and inspected at a FEI Morgagni 268 D transmission electron microscope at 80 kV.

### Measurement of transepithelial resistance

The transepithelial resistance of the hESC-derived RPE cells growing on transwell filters and the empty transwell filters only coated with growth factor-reduced-Matrigel as a control was measured by using an EVOM epithelial voltohmmeter (World Precision Instruments, Hamden, CT) with a pair of chopstick electrodes.

### Phagocytosis analyses using retinal explant co-culture model

The transwell filter (diameter: 6.5 mm) with hESC-derived RPE growing on it was excised and transferred to bigger transwell filter (diameter: 24 mm) with the cells facing up, for easier handling. Neural retinal explants from transgenic mice expressing human RHODOPSIN-GFP [33] were dissected out in PBS and oriented on top of the RPE cell sheet to make the outer segments localize adjacent to the apical surface of hESC-derived RPE. By adding one small drop of low-melting-point agarose gently on top of the retinal explant, the outer segment layer better contacted the RPE. 1 ml of N2B27 medium supplemented with 10% FBS was added to the lower chamber of the transwell so that the medium just touched the surface of the transwell filters. The hESC-derived RPE plus mouse retina composites were fixed in 4% PFA for 1 hour at RT after 24 hours of co-culture, followed by immersion in 30% sucrose at 4°C overnight and subsequent embedding in Tissue-Tek O.C.T.™ Compound (Sakura). The thickness of cross-sections was 10 µm.

### *In situ* hybridization on cyst cryosections

hESC-derived cysts at representative time points were fixed in 4% fresh PFA for 20 min at RT, washed in PBS, equilibrated in 30% sucrose and frozen in Tissue-Tek O.C.T.™ Compound (Sakura). *In situ* hybridization on cryosections of 10 µm thickness was performed as described [46]. Briefly, the sections were washed in PBS/0.1% Tween, hybridized with 500 ng/ml DIG-labeled probe in hybridization buffer (50% formamide, 10% dextran, 5× SSC, 0.1% Tween, 1 mg/ml yeast RNA, 100 µg/ml heparin, 1× Denhardt's, 0.1% CHAPS, 5 mM EDTA) overnight at 70°C. Slides were washed three times an hour and then overnight at 70°C in 5× SSC buffer (50% formamide, 5× SSC, 0.1% Tween), followed by twice an-hour washes at 70°C in post-hybridization buffer (50% formamide, 2× SSC, 0.1% Tween). Slides were further washed twice 5 min and once 20 min at RT in maleic acid buffer (100 mM maleic acid pH 7.5, 150 mM NaCl, 0.1% Tween), blocked in maleic acid buffer plus 1% blocking reagent (Roche) for an hour at RT, and then incubated overnight at 4°C with anti-DIG antibody (1∶5000, Roche) in this blocking solution. Slides were washed 5× 10 min with maleic acid buffer and 2× 10 min with alkaline phosphatase buffer (100 mM Tris pH 9.5, 50 mM MgCl_2_, 100 mM NaCl, 0.1% Tween). Each slide was overlaid with BM purple (Roche) for 6–48 hours at 37°C. The reaction was stopped in cold PBS/1 mM EDTA and the slides were mounted in 50% glycerol.

Sense and antisense probes of *OCT4* and *RX* for *in situ* hybridizations were obtained by RT-PCR from total RNA of undifferentiated or differentiated hESCs. Primers used to amplify the fragments of *OCT4* (database accession no. NM_203289) and *RX* (database accession no. NM_013435) cDNA were shown in Table S2. Sense and antisense probes of *PAX6* were prepared from the Full Length cDNA Clone IRATp970B0617D (imaGenes, database accession no. BC011953).

### Statistical analyses

Values are expressed as means±s.e.m.. For quantification of cysts, 2 to 3 dishes were used for staining per marker at each experiment. Around 100 randomly chosen fields, 100–200 cysts in total, were scored for each marker under confocal microscope. The total number of the cysts was determined by Hoechst nuclear staining. Nuclear localized staining for each marker on all Hoechst-stained nuclei within cysts was manually scored as positive. Both uniform nuclear staining signal of each marker within the cysts or strong signal at the basal-lateral side and weaker signal at the apical side were scored as positive. All experiments were repeated at least 3 times. For quantification of pigmented cells, 1–2 wells were used at each experiment. 3–4 randomly chosen fields, 500–1000 cells in total, were manually counted. The total number of the cells was determined by Hoechst nuclear staining. Experiments were performed in 3 replicates. For the ONL thickness and protected area analyses, 6 RCS rats transplanted with approximately 120,000 human ESC-derived RPE cells, 5 with human fibroblasts, 5 sham-injected and 6 untreated controls were quantified in central (protected) and peripheral regions using approximately every 8^th^ serial section. Areas of experimental retinal sections that showed an ONL with more than three rows of nuclei were defined as rescued allowing calculating the total “protected area” per eye. Statistical analyses were performed with IGOR PRO 6.1.

## Supporting Information

Figure S1 **Specificity of antibodies.** The antibodies used for immunostaining were tested on mouse embryonic sections in the eyefield at representative stages. Inset: higher magnification confirming the nuclear staining of Mitf. Nuclei were counterstained with Hoechst. Scale bar, 50 µm
(TIF)

Figure S2 **Expression of** ***OCT4*** **,** ***PAX6*** **and** ***RX*** **at the RNA level in hESC-derived cysts at representative time points.** (A) *In situ* hybridization of *OCT4*, *PAX6* and *RX*. Day 1 cryosectioned cysts were positive for *OCT4* but negative for *PAX6* and *RX*. Day 5 and Day 15 cryosectioned cysts were positive for *PAX6* and *RX* but negative for *OCT4*. (B) *In situ* hybridization using the *PAX6* sense probe as negative control on cryosectioned cysts. Scale bar, 50 µm.
(TIF)

Figure S3 **RPE determination was dependent on cell seeding density and TGF-β signaling.** (A) Top view of transwell filters showing the appearance of pigmented cells derived from H9 cells at Day 25 at different seeding densities in the presence Activin A (100 ng/ml). Too high or too low cell seeding density failed to induce the formation of a pigmented cell sheet. (B) Differentiation of H1 cells to RPE does not require exogenous Activin A, but does depend on TGF-β/Activin-related signaling. Top view of transwell filters showing the appearance of pigmented cells derived from H1 cells at Day 25 in the presence or absence of SB431542 (8 µM). The TGF-β inhibitor SB431542 completely blocked the pigmentation of cells. Scale bar, 1 mm.
(TIF)

Figure S4 **Differentiation of neural retina progenitor cells from H9 cells.** (A) Schematic of neural retina differentiation protocol. UD: undifferentiated. (B) Immunostaining of CRX (grey), CHX10 (red) and PAX6 (green) at Day 20 (top row), Day 40 (middle row) and Day 60 (bottom row) during neural retina differentiation of H9 cells. The expression of CRX was up-regulated gradually and surrounded by CHX10 and/or PAX6 positive cells in a rosette-like structure by Day 60. (C) Immunostaining of CRX, CHX10 and PAX6 on cryosectioned P7 mouse retina. P7: postnatal day 7. CRX, CHX10 and PAX6 were expressed with a layered pattern *in vivo*. Nuclei were counterstained with Hoechst. Scale bar, 50 µm.
(TIF)

Figure S5 **Differentiation of RPE cells from human iPSCs.** (A) A phase contrast image of human iPSC-derived cysts at Day 5. (B) Top view of transwell filters showing the appearance of pigmented cells derived from human iPSCs at Day 30 in the presence or absence of Activin A (100 ng/ml). (C) Human iPSC-derived cysts were positive for PAX6 (upper row green), RX (upper row red) and OTX2 (lower row green) at Day 5. The expression level of Pax6 was low. ZO-1 (lower row red) was expressed toward the apical side of the cysts indicating polarized epithelial phenotype. Nuclei were counterstained with Hoechst. Scale bars, 50 µm (A, C), 1 mm (B).
(TIF)

Figure S6 **Micrographs of RCS rat retinal transplantation injection sites and untreated retinas.** (A) A merged immunofluorescence image of human b2 microglobulin (red) and Dapi (blue) in the central region of hESC-derived RPE cell transplanted retina. Donor cells (red) were identified by human-specific antibody. Inset: higher magnification of injection site and adjacent region to the injection site, respectively. Around the injection site hESC-derived RPE cells formed clusters and the underlying ONL showed disruption and rosette formation (arrow head). Adjacent to the injection site, donor cells integrated into the host RPE monolayer, generating monolayer-like structures with the ONL underneath well preserved. (B) Electron microscopic analyses of transplanted RPE cells. Neighboring donor cells were connected via tight junctions/desmosomes (arrows) (C, D) Representative fluorescence images of central or peripheral region in the untreated retina. Dashed box showed the ONL. Untreated RCS controls displayed 1–2 rows of nuclei in ONL both in central (C) and peripheral (D) regions of the retina. GCL: ganglion cell layer, INL: inner nuclear layer, ONL: outer nuclear layer. Nuclei were counterstained with Dapi. Scale bars, 200 µm (A), 1 µm (B), 20 µm (C, D).
(TIF)

Movie S1 **A time-lapse movie showing cyst formation from hESC clumps within 24 hours after embedding in Matrigel.**
(MOV)

Movie S2 **A movie of serial confocal images through z-axis showing GFP-labeled outer segments (green) inside hESC-derived pigmented cells in the retinal explant co-culture model.** Each image of the z-stack was merged of RHODOPSIN-GFP (green), human nuclei (red), and Hoechst (blue) images.
(MOV)

Table S1 **Published protocols of RPE generation from human pluripotent stem cells.**
(DOCX)

Table S2 **List of primers used for RT-PCR and** ***in situ*** **hybridization.**
(DOCX)
