# Supplementary figures and images for: Three-Dimensional Neuroepithelial Culture from Human Embryonic Stem Cells and Its Use for Quantitative Conversion to Retinal Pigment Epithelium
Source: PLoS One. 2013 Jan 24;8(1):e54552. doi: 10.1371/journal.pone.0054552 (PMC3554725; doi:10.1371/journal.pone.0054552)

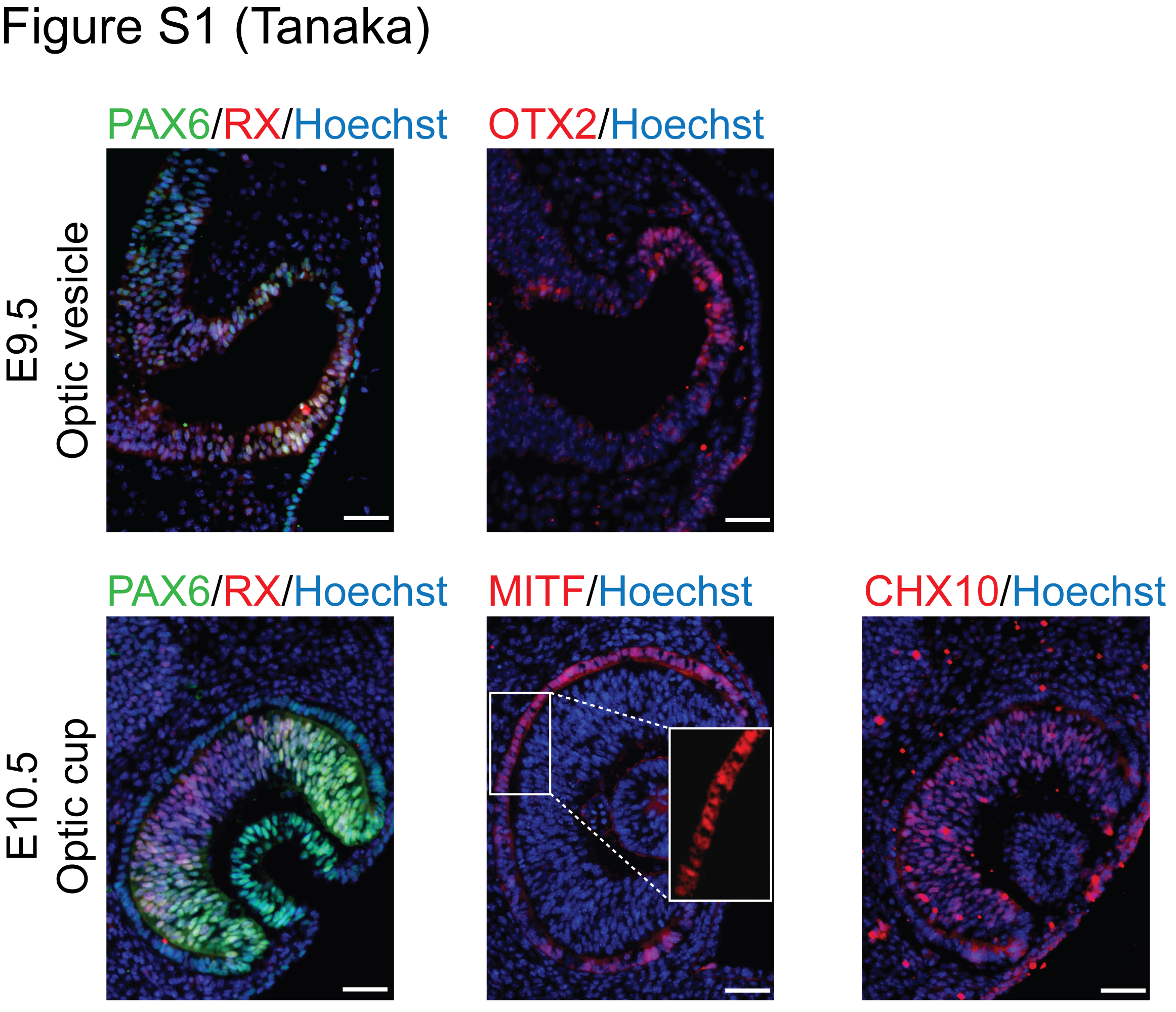

Supplement: Figure S1 — Specificity of antibodies. The antibodies used for immunostaining were tested on mouse embryonic sections in the eyefield at representative stages. Inset: higher magnification confirming the nuclear staining of Mitf. Nuclei were counterstained with Hoechst. Scale bar, 50 µm (TIF) [file pone.0054552.s001.tif]

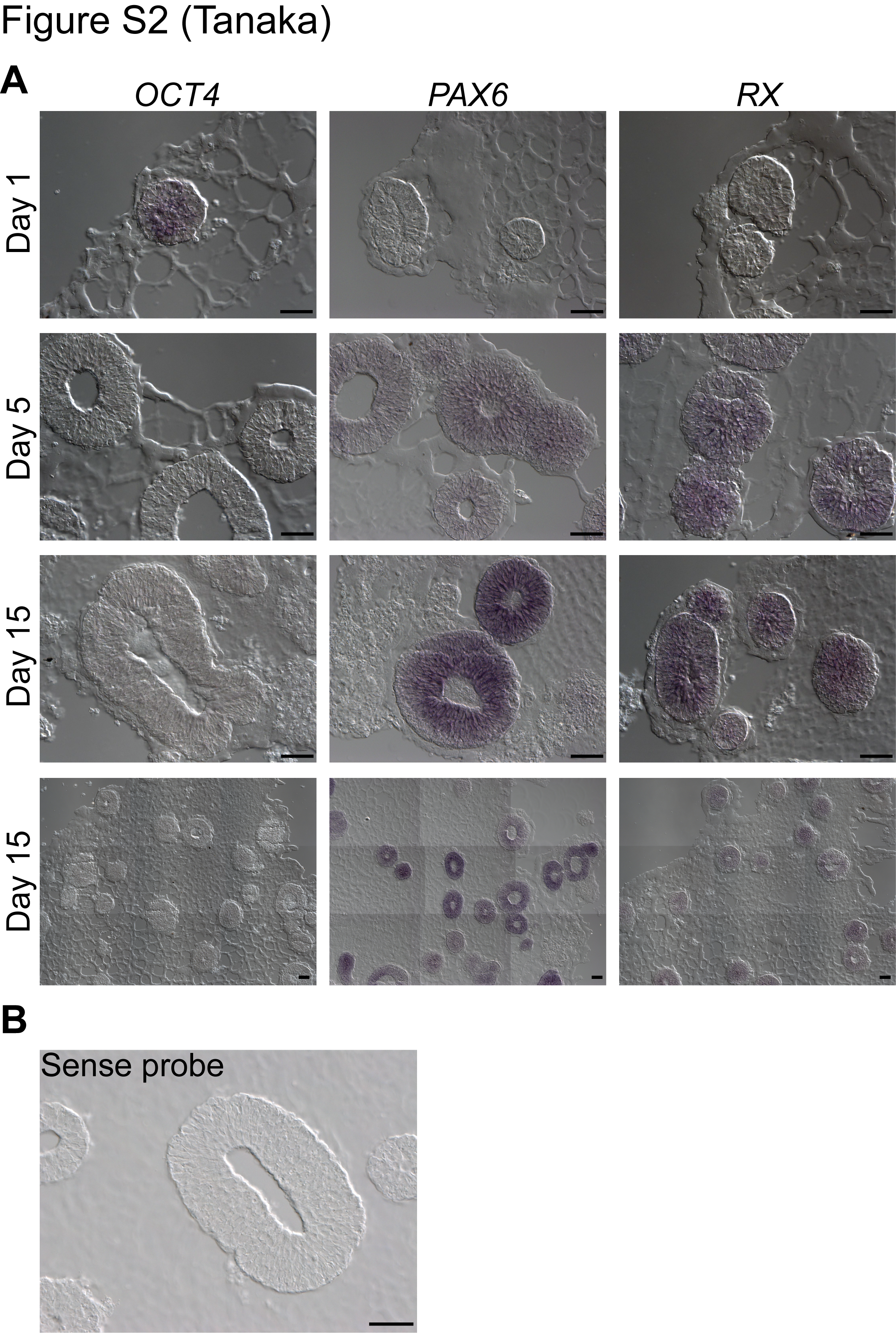

Supplement: Figure S2 — Expression of OCT4 , PAX6 and RX at the RNA level in hESC-derived cysts at representative time points. (A) In situ hybridization of OCT4, PAX6 and RX. Day 1 cryosectioned cysts were positive for OCT4 but negative for PAX6 and RX. Day 5 and Day 15 cryosectioned cysts were positive for PAX6 and RX but negative for OCT4. (B) In situ hybridization using the PAX6 sense probe as negative control on cryosectioned cysts. Scale bar, 50 µm. (TIF) [file pone.0054552.s002.tif]

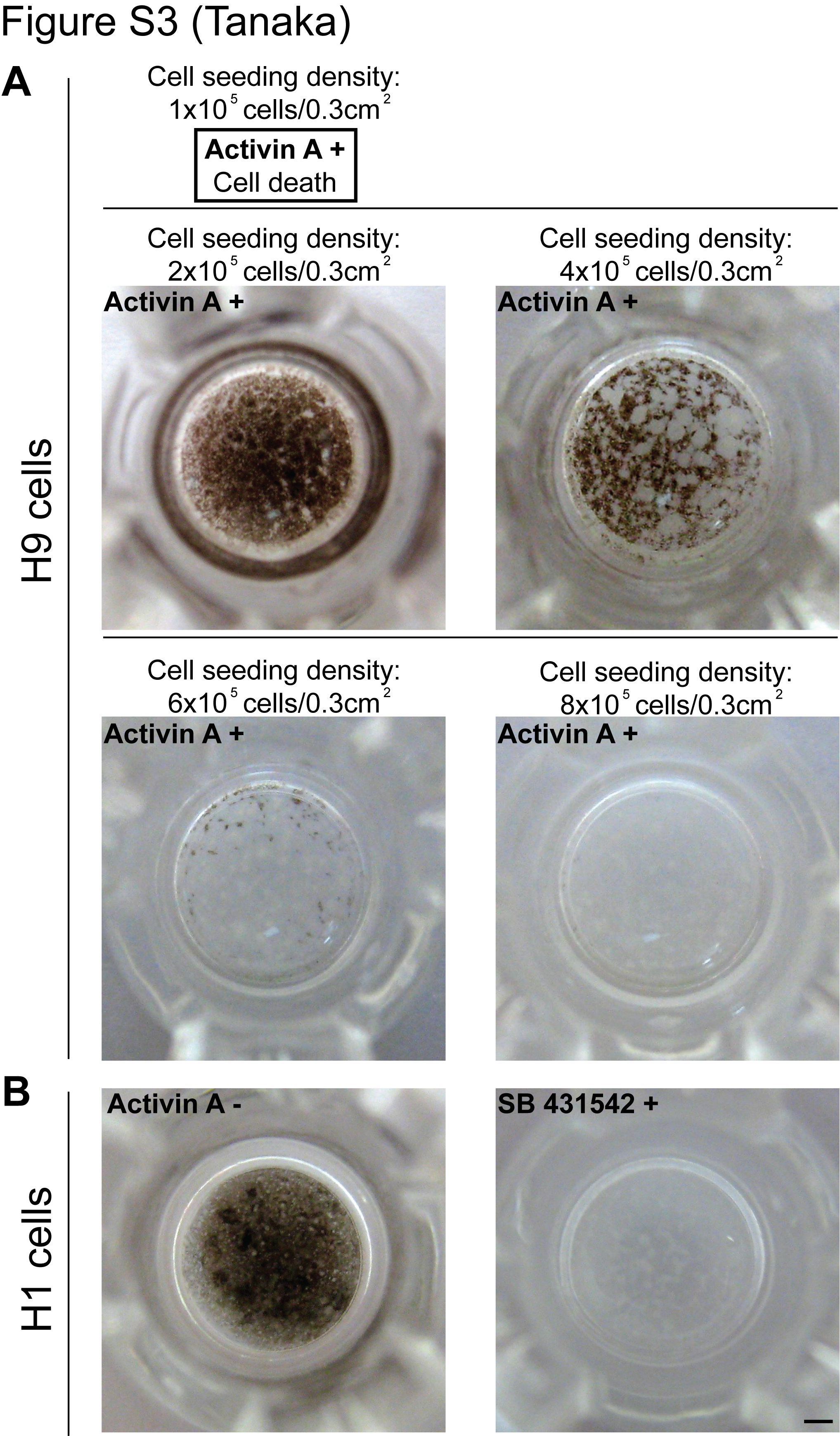

Supplement: Figure S3 — RPE determination was dependent on cell seeding density and TGF-β signaling. (A) Top view of transwell filters showing the appearance of pigmented cells derived from H9 cells at Day 25 at different seeding densities in the presence Activin A (100 ng/ml). Too high or too low cell seeding density failed to induce the formation of a pigmented cell sheet. (B) Differentiation of H1 cells to RPE does not require exogenous Activin A, but does depend on TGF-β/Activin-related signaling. Top view of transwell filters showing the appearance of pigmented cells derived from H1 cells at Day 25 in the presence or absence of SB431542 (8 µM). The TGF-β inhibitor SB431542 completely blocked the pigmentation of cells. Scale bar, 1 mm. (TIF) [file pone.0054552.s003.tif]

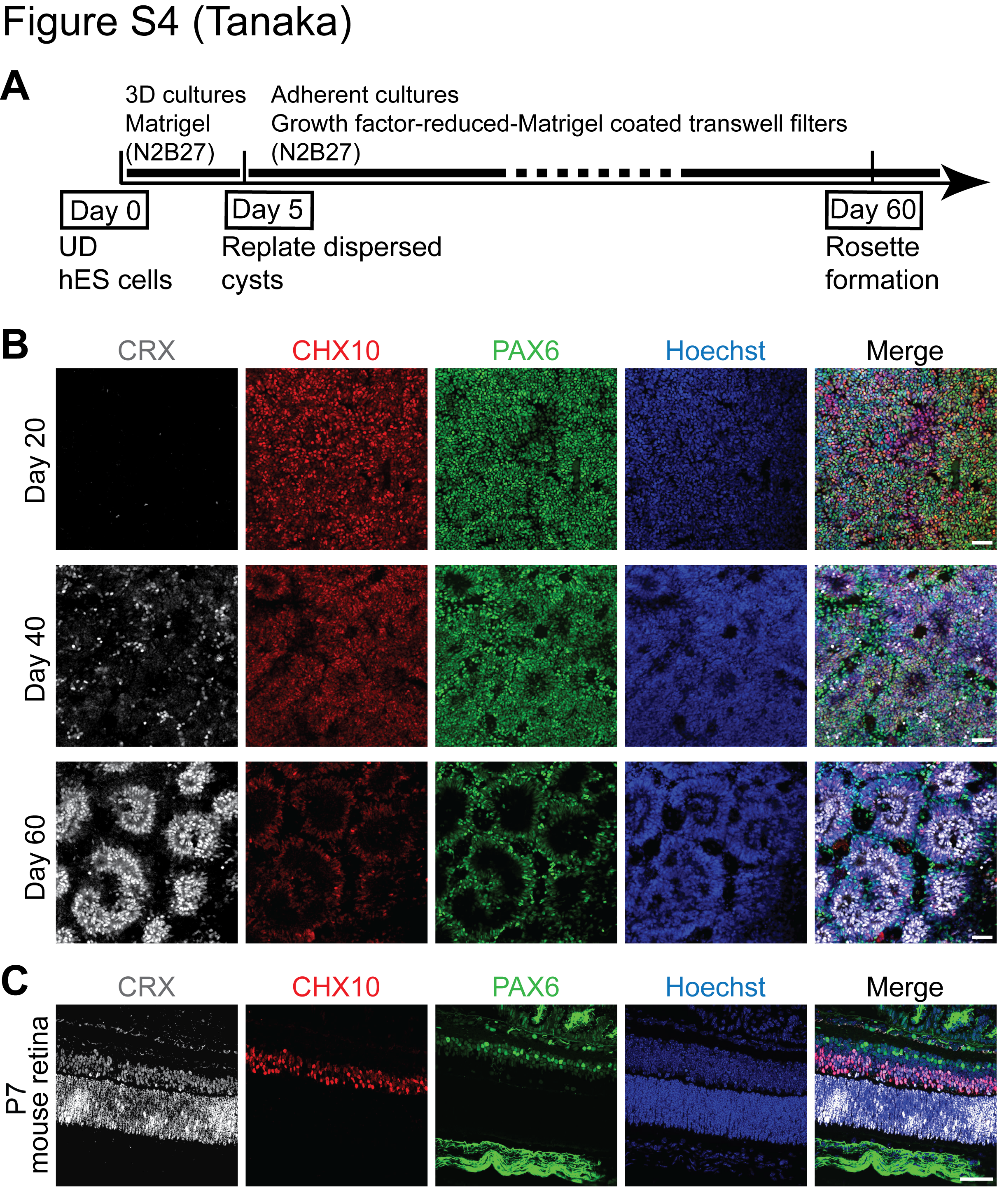

Supplement: Figure S4 — Differentiation of neural retina progenitor cells from H9 cells. (A) Schematic of neural retina differentiation protocol. UD: undifferentiated. (B) Immunostaining of CRX (grey), CHX10 (red) and PAX6 (green) at Day 20 (top row), Day 40 (middle row) and Day 60 (bottom row) during neural retina differentiation of H9 cells. The expression of CRX was up-regulated gradually and surrounded by CHX10 and/or PAX6 positive cells in a rosette-like structure by Day 60. (C) Immunostaining of CRX, CHX10 and PAX6 on cryosectioned P7 mouse retina. P7: postnatal day 7. CRX, CHX10 and PAX6 were expressed with a layered pattern in vivo. Nuclei were counterstained with Hoechst. Scale bar, 50 µm. (TIF) [file pone.0054552.s004.tif]

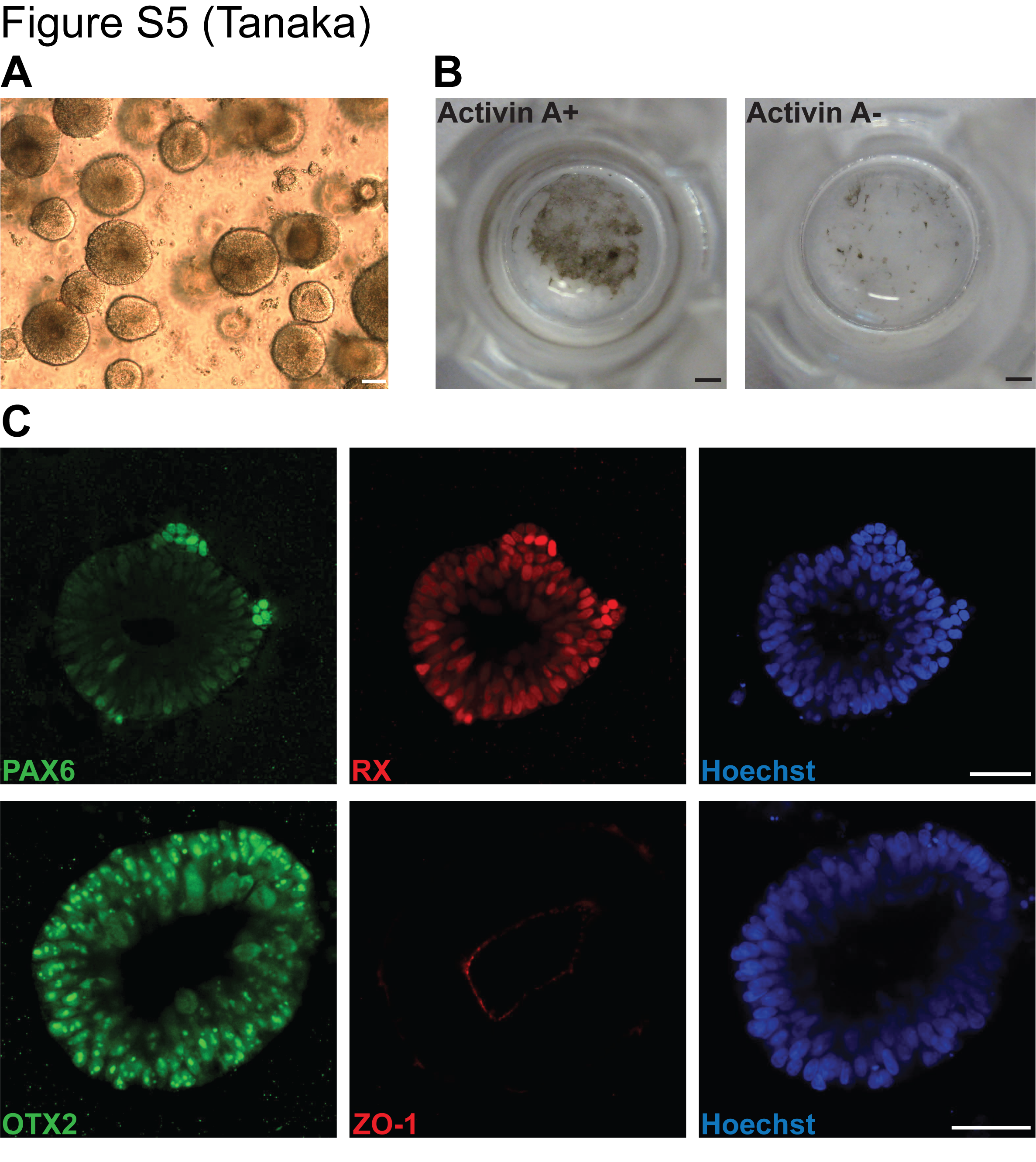

Supplement: Figure S5 — Differentiation of RPE cells from human iPSCs. (A) A phase contrast image of human iPSC-derived cysts at Day 5. (B) Top view of transwell filters showing the appearance of pigmented cells derived from human iPSCs at Day 30 in the presence or absence of Activin A (100 ng/ml). (C) Human iPSC-derived cysts were positive for PAX6 (upper row green), RX (upper row red) and OTX2 (lower row green) at Day 5. The expression level of Pax6 was low. ZO-1 (lower row red) was expressed toward the apical side of the cysts indicating polarized epithelial phenotype. Nuclei were counterstained with Hoechst. Scale bars, 50 µm (A, C), 1 mm (B). (TIF) [file pone.0054552.s005.tif]

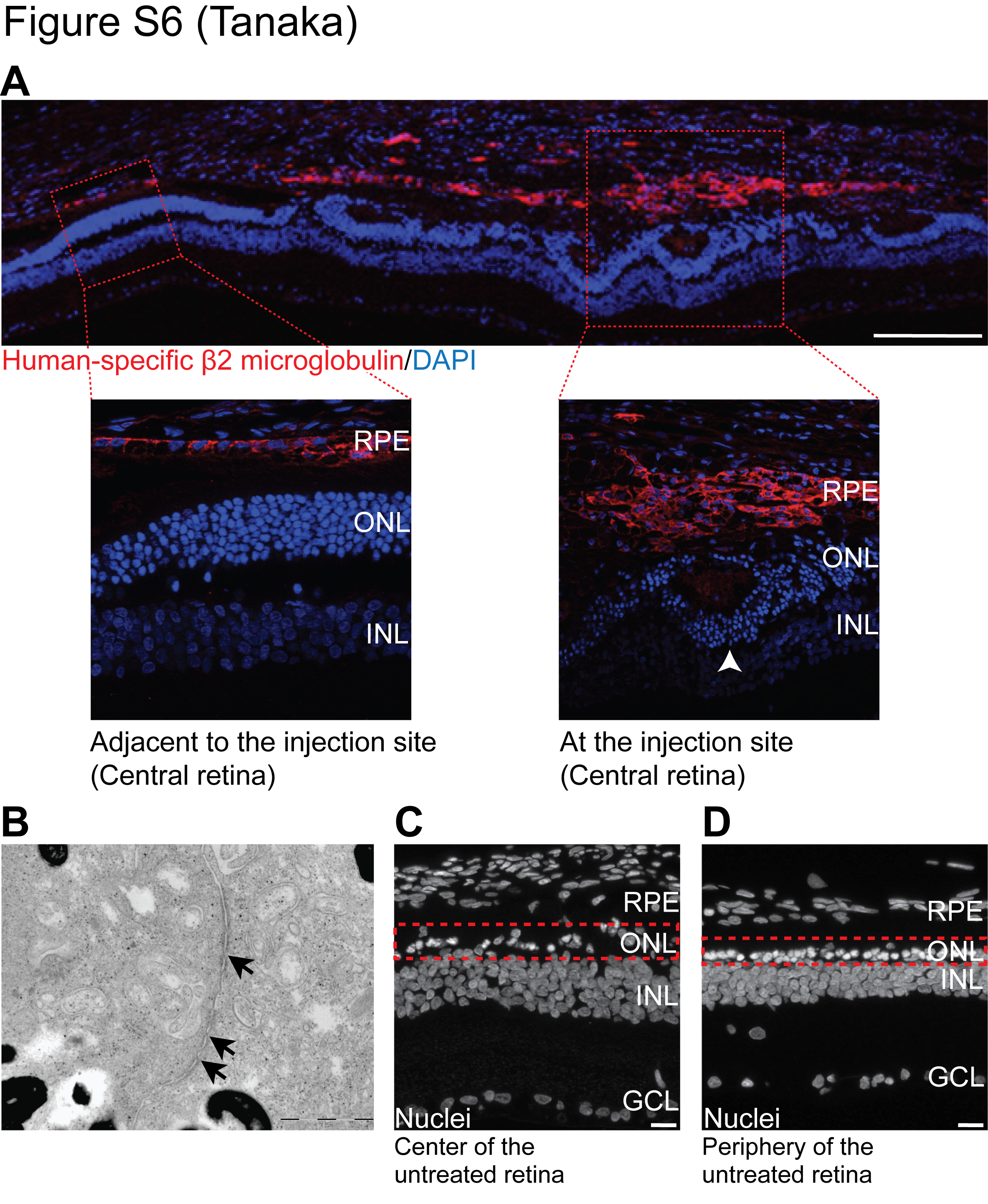

Supplement: Figure S6 — Micrographs of RCS rat retinal transplantation injection sites and untreated retinas. (A) A merged immunofluorescence image of human b2 microglobulin (red) and Dapi (blue) in the central region of hESC-derived RPE cell transplanted retina. Donor cells (red) were identified by human-specific antibody. Inset: higher magnification of injection site and adjacent region to the injection site, respectively. Around the injection site hESC-derived RPE cells formed clusters and the underlying ONL showed disruption and rosette formation (arrow head). Adjacent to the injection site, donor cells integrated into the host RPE monolayer, generating monolayer-like structures with the ONL underneath well preserved. (B) Electron microscopic analyses of transplanted RPE cells. Neighboring donor cells were connected via tight junctions/desmosomes (arrows) (C, D) Representative fluorescence images of central or peripheral region in the untreated retina. Dashed box showed the ONL. Untreated RCS controls displayed 1–2 rows of nuclei in ONL both in central (C) and peripheral (D) regions of the retina. GCL: ganglion cell layer, INL: inner nuclear layer, ONL: outer nuclear layer. Nuclei were counterstained with Dapi. Scale bars, 200 µm (A), 1 µm (B), 20 µm (C, D). (TIF) [file pone.0054552.s006.tif]
