# Supplementary material for: Three-Dimensional Neuroepithelial Culture from Human Embryonic Stem Cells and Its Use for Quantitative Conversion to Retinal Pigment Epithelium
Source: PLoS One. 2013 Jan 24;8(1):e54552. doi: 10.1371/journal.pone.0054552 (PMC3554725; doi:10.1371/journal.pone.0054552)
Supplement: Table S1 — Published protocols of RPE generation from human pluripotent stem cells. (DOCX) [file pone.0054552.s009.docx]

| **Manuscript** | **Stem cell source** | **RPE or RPC** | **Essential method** | **Time** | **Efficiency** |
| --- | --- | --- | --- | --- | --- |
| [[1](#_ENREF_1)] | Human, ESC | RPE | Spontaneous differentiation | 4-8 weeks | Small fraction ~1% pigmentation |
| [2] | Human, ESC | RPE | Spontaneous differentiation | ~8 weeks | Pigmented monolayer cell sheets of several millimeters across |
| [[3](#_ENREF_3)] | Human, iPSC | RPE | Spontaneous differentiation | 60 days | >1% pigmentation |
| [[4](#_ENREF_4)] | Human, ESC | RPE | Floating aggregates +Activin A and NIC | 6 weeks | 33% pigmentation |
| [[5](#_ENREF_5),[6](#_ENREF_6)] | Human, ESC, iPSC | RPC, RPE | Floating aggregates | 16 days  40 days | >95% RX^+^, 25% MITF^+^ |
| [[7](#_ENREF_7)] | Primate, ESC | RPC, RPE | Floating aggregate +DKK1 and Lefty (hESC) | 35 days  50 days 120 days | 16% RX^+^-PAX6^+^ 31% MITF­^+^-PAX6^+^ 34% ZO1^+^ |

**Table S1.** Published protocols of RPE generation from human pluripotent stem cells.

1. Klimanskaya I, Hipp J, Rezai KA, West M, Atala A, et al. (2004) Derivation and comparative assessment of retinal pigment epithelium from human embryonic stem cells using transcriptomics. Cloning Stem Cells 6: 217-245.

2. Vugler A, Carr AJ, Lawrence J, Chen LL, Burrell K, et al. (2008) Elucidating the phenomenon of HESC-derived RPE: anatomy of cell genesis, expansion and retinal transplantation. Exp Neurol 214: 347-361.

3. Buchholz DE, Hikita ST, Rowland TJ, Friedrich AM, Hinman CR, et al. (2009) Derivation of functional retinal pigmented epithelium from induced pluripotent stem cells. Stem Cells 27: 2427-2434.

4. Idelson M, Alper R, Obolensky A, Ben-Shushan E, Hemo I, et al. (2009) Directed differentiation of human embryonic stem cells into functional retinal pigment epithelium cells. Cell Stem Cell 5: 396-408.

5. Meyer JS, Howden SE, Wallace KA, Verhoeven AD, Wright LS, et al. (2011) Optic vesicle-like structures derived from human pluripotent stem cells facilitate a customized approach to retinal disease treatment. Stem cells 29: 1206-1218.

6. Meyer JS, Shearer RL, Capowski EE, Wright LS, Wallace KA, et al. (2009) Modeling early retinal development with human embryonic and induced pluripotent stem cells. Proc Natl Acad Sci U S A 106: 16698-16703.

7. Osakada F, Ikeda H, Mandai M, Wataya T, Watanabe K, et al. (2008) Toward the generation of rod and cone photoreceptors from mouse, monkey and human embryonic stem cells. Nat Biotechnol 26: 215-224.
