# Supplementary material for: Three-Dimensional Neuroepithelial Culture from Human Embryonic Stem Cells and Its Use for Quantitative Conversion to Retinal Pigment Epithelium
Source: PLoS One. 2013 Jan 24;8(1):e54552. doi: 10.1371/journal.pone.0054552 (PMC3554725; doi:10.1371/journal.pone.0054552)
Supplement: Table S2 — List of primers used for RT-PCR and in situ hybridization. (DOCX) [file pone.0054552.s010.docx]

|  | **Gene** |  | **Primer** |
| --- | --- | --- | --- |
| RT-PCR | OCT4 | Forward | 5’-AGTGAGAGGCAACCTGGAGA-3’ |
|  |  | Reverse | 5’-GTGAAGTGAGGGCTCCCATA-3’ |
|  | SOX1 | Forward | 5’-CAATGCGGGGAGGAGAAGTC-3’ |
|  |  | Reverse | 5’-CTCTGGACCAAACTGTGGCG-3’ |
|  | PAX6 | Forward | 5’-AACAGACACAGCCCTCACAAACA-3’ |
|  |  | Reverse | 5’-CGGGAACTTGAACTGGAACTGAC-3’ |
|  | RX | Forward | 5’-GAATCTCGAAATCTCAGCCC-3’ |
|  |  | Reverse | 5’-CTTCACTAATTTGCTCAGGAC-3’ |
|  | SIX3 | Forward | 5’-ACCACAAGTTCACCAAGGAGTCTC-3’ |
|  |  | Reverse | 5’-ATTCCGAGTCGCTGGAGGTTAC-3’ |
|  | MITF | Forward | 5’-GACAGAAGAAACTGGAGCACGC-3’ |
|  |  | Reverse | 5’-TCCGAGGTTGTTGTTGAAGGTG-3’ |
|  | CHX10 | Forward | 5’-GCTCGGATTCTGAAGATGTTTCC-3’ |
|  |  | Reverse | 5’-TGCCTCCAGCGACTTTTTGTG-3’ |
|  | CRX | Forward | 5’-ATGATGGCGTATATGAACCC-3’ |
|  |  | Reverse | 5’-TCTTGAACCAAACCTGAACC-3’ |
|  | BF-1 | Forward | 5’-ACTCAAAACTCGCTGGGCAAC-3’ |
|  |  | Reverse | 5’-CGTGGGGGAAAAAGTAACTGG-3’ |
|  | PAX2 | Forward | 5´-ATGTTCGCCTGGGAGATTCG-3´ |
|  |  | Reverse | 5´-GCAAGTGCTTCCGCAAACTG-3´ |
|  | HOX B1 | Forward | 5´-TCAGAAGGAGACGGAGGCTA-3´ |
|  |  | Reverse | 5´-GTGGGGGTGTTAGGTTCTGA-3´ |
|  | HOX C5 | Forward | 5´-TCGGGGTGCTTCCTTGTAGC-3´ |
|  |  | Reverse | 5´-TTCGTGGCAGGGACTATGGG-3´ |
|  | GAPDH | Forward | 5’-GGGGAGCCAAAAGGGTCATCATCT-3’ |
|  |  | Reverse | 5’-GAGGGGCCATCCACAGTCTTCT-3’ |
| *In situ* hybridization | OCT4 | Forward | 5’-AAGGATGTGGTCCGAGTGTGGTTC-3’ |
|  |  | Reverse | 5’-TAGAAGGGCAGGCACCTCAGTTTG-3’ |
|  | RX | Forward | 5’-AATCGTCCCCATTCCGAACG-3’ |
|  |  | Reverse | 5’-TGGTCATCCTTTTCCCAAGTCG-3’ |

**Table S2.** List of primers used for RT-PCR and *in situ* hybridization.
